# Collection and Chemical Composition of Phloem Sap from *Citrus sinensis* L. Osbeck (Sweet Orange)

**DOI:** 10.1371/journal.pone.0101830

**Published:** 2014-07-11

**Authors:** Faraj Hijaz, Nabil Killiny

**Affiliations:** Citrus Research and Education Center, University of Florida, IFAS, Lake Alfred, Florida, United States of America; Institute of Vegetables and Flowers, Chinese Academy of Agricultural Science, China

## Abstract

Through utilizing the nutrient-rich phloem sap, sap feeding insects such as psyllids, leafhoppers, and aphids can transmit many phloem-restricted pathogens. On the other hand, multiplication of phloem-limited, uncultivated bacteria such as *Candidatus* Liberibacter asiaticus (*C*Las) inside the phloem of citrus indicates that the sap contains all the essential nutrients needed for the pathogen growth. The phloem sap composition of many plants has been studied; however, to our knowledge, there is no available data about citrus phloem sap. In this study, we identified and quantified the chemical components of phloem sap from pineapple sweet orange. Two approaches (EDTA enhanced exudation and centrifugation) were used to collect phloem sap. The collected sap was derivatized with methyl chloroformate (MCF), N-methyl-N- [tert-butyl dimethylsilyl]-trifluroacetamide (MTBSTFA), or trimethylsilyl (TMS) and analyzed with GC-MS revealing 20 amino acids and 8 sugars. Proline, the most abundant amino acid, composed more than 60% of the total amino acids. Tryptophan, tyrosine, leucine, isoleucine, and valine, which are considered essential for phloem sap-sucking insects, were also detected. Sucrose, glucose, fructose, and inositol were the most predominant sugars. In addition, seven organic acids including succinic, fumaric, malic, maleic, threonic, citric, and quinic were detected. All compounds detected in the EDTA-enhanced exudate were also detected in the pure phloem sap using centrifugation. The centrifugation technique allowed estimating the concentration of metabolites. This information expands our knowledge about the nutrition requirement for citrus phloem-limited bacterial pathogen and their vectors, and can help define suitable artificial media to culture them.

## Introduction

Plant phloem sap is rich in nutrients [1]; it contains high quantities of sugars, amino acids, organic acids, vitamins, and inorganic ions. Because phloem sap is rich in nutrients and free of feeding deterrents and toxins, it is exclusively consumed by many phloem sap feeding insects [2] which facilitates transmission of several vector-borne plant pathogens [3]. Examples of economically important citrus diseases, such as huanglongbing (HLB), citrus tristeza, and citrus stubborn are caused by phloem sap-limited pathogens and are transmitted by piercing-sucking insects [3].

Studies on phloem sap composition investigated the nutrient intake and allocation in plants [4], [5], while others were conducted to investigate the effect of changes in phloem sap composition on insect feeding behavior [6], insect symbiont metabolism, and insect honeydew composition [7]. In addition, some studies addressed the relation between phloem sap and insect honeydew composition to explain why some insect vectors have an expanded range of host plants which have similar phloem sap composition [8]. Phloem sap composition was also used as a tool to assess plant health [9].

Several methods have been used to collect and analyze phloem sap [1], [4]. The incision method is based on spontaneous bleeding of the phloem sap. This method is easy and fast, but it does not work with most plants because the rapid accumulation of callose and P-protein in the sieve plates after incision stops the phloem bleeding [1], [4]. The EDTA method uses ethylenediaminetetraacetic acid (EDTA) to prevent accumulation of callose and P-protein in the phloem sieve. Briefly, the tip of the petioles or a piece of bark tissue is immersed in EDTA solution for a few hours to collect the soluble amino acids, sugars, and other metabolites to be analyzed. The EDTA-exudation method is easy, allows to collect a large quantity of phloem exudates, and works with most plants. However, it does not measure the concentration of the phloem sap content. Moreover, phloem exudates would be contaminated with xylem sap and substances from other tissues. In the stylectomy method, the insect stylet is cut while the insect feeds on the host plant and the exudate is collected using a microcapillary. Although this method yields a pure phloem sap, the amount obtained by this method is small and accurate estimate of chemical composition is not guaranteed. In addition, this method is not applicable to all insect species and it is restricted to young plants [1].

Phloem sap is a complex mixture of organic and inorganic substances [10]. A previous study has shown that sugars and amino acids are the most predominant metabolites in phloem sap [1]. Sucrose is the main sugar in phloem sap and its concentration varies between species. Other sugars such as hexoses (e.g., glucose and fructose), raffinose-oligosaccharides, and polyols (mannitol and sorbitol) are also found in phloem sap [1]. Amino acids are the main form of reduced nitrogen in phloem sap, and their relative and total concentration varies among species [1]. Organic acids such as malic, succinic, ascorbic, and citric acid are present in many species [1].

Citrus is an important crop that grows in areas starting at ±40° latitude [11]. The US and Brazil are the major citrus producers in the world [11]. A wide range of insects, bacteria, and viruses may challenge citrus production. Among pathogens, *Spiroplasma citri*, citrus tristeza virus (CTV), and *Candidatus* Liberibacter asiaticus (*C*Las) transmitted by piercing-sucking insects [3].

Huanglongbing (HLB), also called citrus greening disease, is a major disease affecting billions of dollars of loss in citrus industries worldwide. *Candidatus* Liberibacter asiaticus (*C*Las), which is associated with HLB, is a phloem-restricted and uncultivable gram-negative bacterium [12]. Three species of *Candidatus* Liberibacter have been associated with HLB: *Candidatus* Liberibacter asiaticus (Asia, North America, and Brazil), *Candidatus* Liberibacter africanus (*C*Laf) (Africa), and *Candidatus* Liberibacter americanus (*C*Lam) (Brazil) [13]. *C*Las and *C*Lam are naturally transmitted by the ACP, *Diaphorina citri* Kuwayama (Hemiptera: Psyllidae) while *C*Laf is transmitted by the African citrus psyllid *Trioza erytrea* (Del Guercio) (Hemiptera: Triozidae) [14].

Although *C*Las grows and multiplies inside its host phloem sap, it cannot be isolated [3]. The growth of this bacterium in its host plant indicates that the phloem sap contains all the essential nutrients needed for its growth. Likewise, the vector, ACP multiplies quickly while feeding on its host plants, but they could not survive for a long when reared on an artificial diet system [15].

The characterization of phloem sap composition from citrus may reveal methods to cultivate *C*Las and improve the artificial diet system for piercing-sucking vectors. Culturing *C*Las is critically important in order to study the pathogen subsequently assisting in finding approaches to limit transmission and development of *C*Las. In addition, development of an artificial diet solution based on the phloem sap composition will enable researchers to study pathogen-insect interactions in absence of plant variable [15].

In the current study, we described the chemical composition of phloem sap from citrus plants. In addition to the EDTA enhanced exudation, we used a new method to collect pure phloem sap that allows us to estimate the real concentration of phloem sap compounds. Furthermore, three different derivatization reagents were used to ensure the detection of most phloem compounds.

## Results and Discussion

The pineapple sweet orange was chosen for this study because it is a suitable host for ACP and *C*Las [16], [17]. To estimate the concentration (in molarity) of metabolites, in addition to the EDTA-enhanced exudation technique, pure phloem sap was obtained by centrifugation. This centrifugation technique has been used previously to collect citrus xylem sap [18].

### Phloem sap pH, °Brix value, and total amino acid content

The average pH of the sap obtained by the centrifugation method was 6.04±0.16 (n = 10) and for that obtained by the EDTA-enhanced exudation technique was 6.03±0.12 (n = 10). These results together showed that the citrus phloem sap is slightly acidic. The literature provides little data about the pH of phloem sap. However, it was believed that phloem sap is moderately alkaline (7.3–8.5) and the acidic pH values reported in the past was likely due to contamination with xylem exudates and bark tissue [1]. In the current study, the xylem tissues were removed before the phloem sap was collected, thus contamination with xylem sap was avoided. Therefore, we believe that the high amount of organic acids is responsible for the slight acidity of citrus phloem sap pH. The fact that *C*Las pathogenic bacterium grows inside the acidic citrus fruit [19] supports our current finding about the acidity of the phloem sap. In addition, the pH of the sap reported in this study is in agreement with the fact that the majority of insects have a slightly acidic hemolymph [20]. This may explain in part why *C*Las grows in both ACP hemolymph and citrus phloem sap [3].

The °Brix value (an estimation of the soluble solid content (SSC)) of pure sap obtained by centrifugation was 9.4±1.3 (n = 10), while it was 10.73±0.22 for phloem sap collected by the EDTA-enhanced exudation technique. This value is close to the °Brix value of citrus juice (5.4–10) [21]. These findings indicate that the citrus phloem sap, similarly to citrus juice, is rich in sugars. The °Brix value of phloem sap is close to that of grapevine (4.5–24) [22]. Because the °Brix value is an estimation of the total dissolved solids including sugars and other soluble organic acids, it is generally higher than the total sugars, and the difference between the °Brix values and the sugar content depends on the amount of other soluble organic acids.

The total amino acids concentration in the phloem sap was also estimated using the ninhydrin method. The total amino acid concentration in sap obtained by centrifugation was 43.6±6.6 mM (n = 12) and for that obtained using EDTA was 29.9±2.25 mM/kg fresh weight bark tissue. Total amino acid concentration in the sap was higher than that found in citrus juice (21 mM) [23].

### Chemical composition of citrus phloem sap

**Figure 1:**
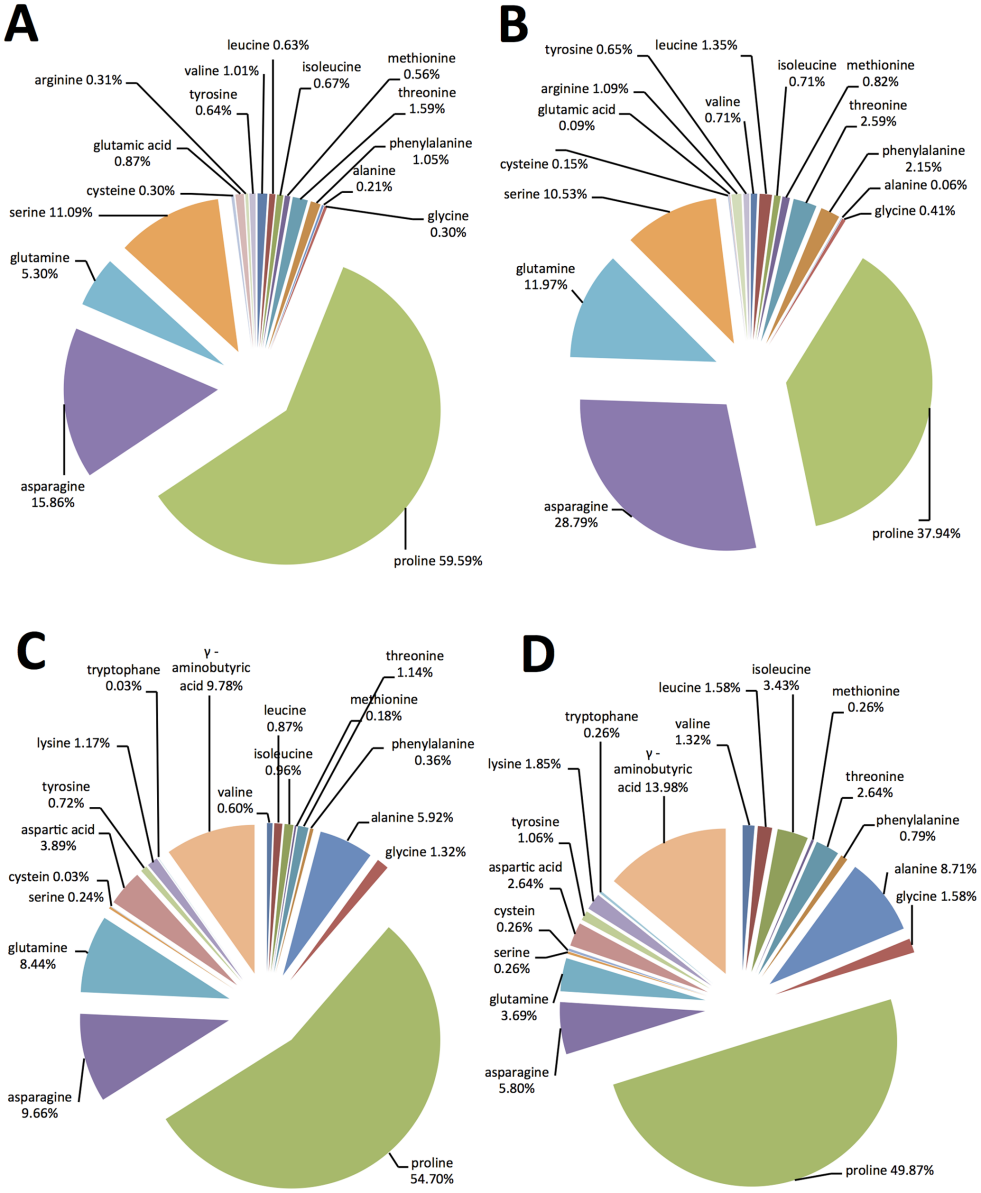
Percentage composition of amino acids in the phloem sap from pineapple sweet orange: A) Phloem sap collected by centrifugation and derivatized with MTBSTFA, B) Phloem sap collected by EDTA exudation and derivatized with MTBSTFA, C) Phloem sap collected by centrifugation and derivatized with MCF, and D) Phloem sap collected by EDTA exudation and derivatized with MCF.

**Figure 2:**
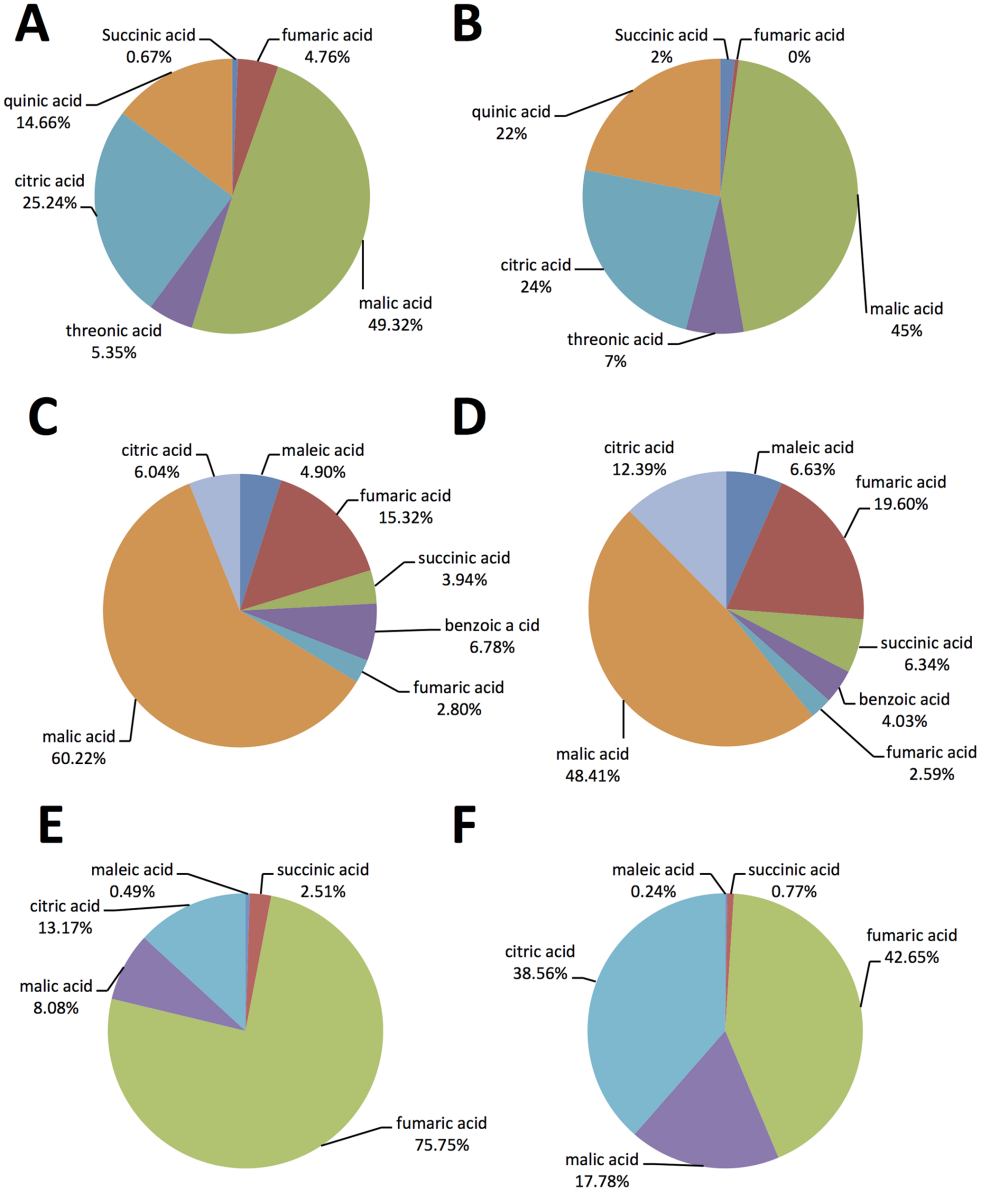
Percentage composition of organic acids in the phloem sap from pineapple sweet orange: A) Phloem sap collected by centrifugation and derivatized with TMS, B) Phloem sap collected by EDTA exudation and derivatized with TMS, C) Phloem sap collected by centrifugation and derivatized with MCF, D) Phloem sap collected by EDTA exudation and derivatized with MCF, E) Phloem sap collected by centrifugation and derivatized with MTBSTFA, and F) Phloem sap collected by EDTA exudation and derivatized with MTBSTFA.

**Figure 3:**
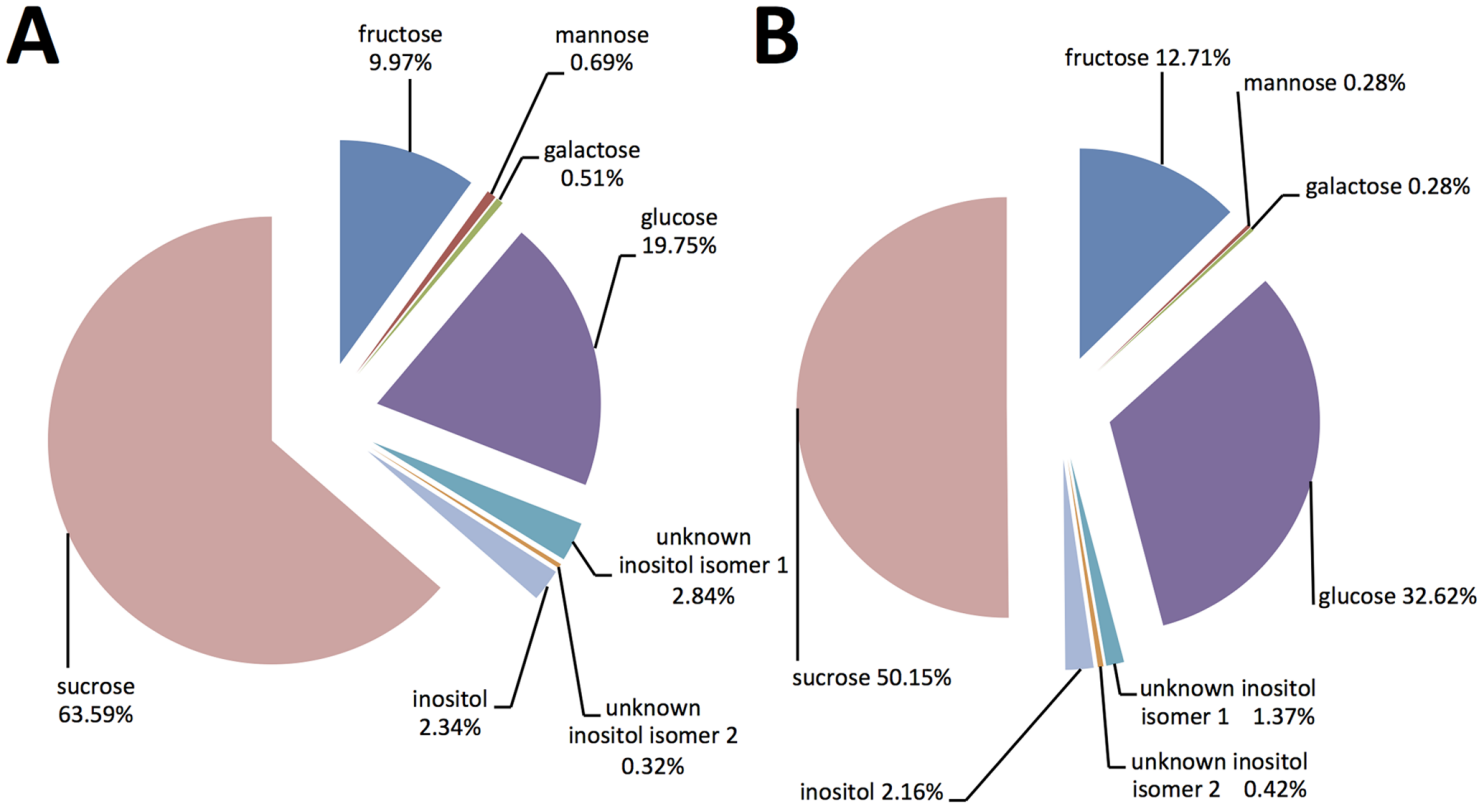
Percentage composition of sugars in the phloem sap from pineapple sweet orange: A) Phloem sap collected by centrifugation and derivatized with TMS and B) Phloem sap collected by EDTA exudation and derivatized with TMS.

**Figure 4:**
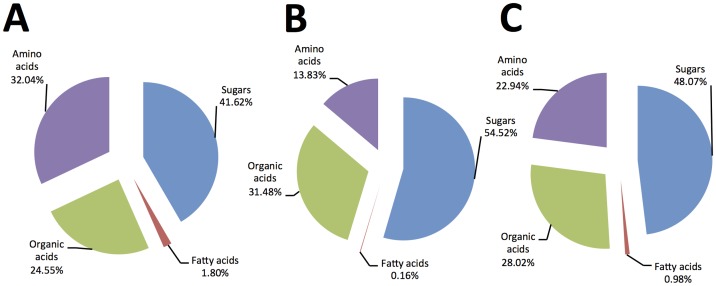
Chemical composition of pineapple sweet orange expressed as percentage composition of the major groups (averages of the different derivatization methods): A) Percentage composition of the phloem sap prepared by centrifugation method and B) Percentage composition of the the phloem sap prepared by EDTA. C) Percentage composition of phloem sap (averages from A and B).

The phloem sap was analyzed by GC-MS after being derivatized by MCF, MTBSTFA, or TMS. More than one derivatization method was used to maximize the number of detected compounds. The concentrations of the metabolites detected in the phloem sap of pineapple sweet orange are shown in Table 1, Table 2, and Table 3. All metabolites detected in the EDTA exudates were also detected in the phloem sap obtained by the centrifugation method. Similarity in compound percentages was found in both collection methods (Figure 1, Figure 2, Figure 3, and Figure 4). Because the centrifugation method results in pure sap, compound concentrations were calculated by molarity (mM). In the EDTA exudation method, we calculated the compound concentrations by m mol/Kg of fresh bark. Variability in results perhaps due to from low sample volume, low metabolite concentration, variation in the phloem sap within plants, and chemical derivatization.

**Table 1:** Amino acids and organic acids detected in pineapple sweet orange phloem sap by GC-MS after MCF derivatization (n = 5).

Compound	LRI	Centrifuge-exudate (mM)	EDTA-enhanced exudate	Centrifuge-Exudate (%)	EDTA-enhanced exudate (%)
			(m mole/Kg tissue)		
**Maleic acid**	1002	1.33±0.34	0.23±0.09	2.22±0.57	3.21±1.26
**Fumaric acid**	1003	4.16±0.59	0.68±0.14	6.96±0.99	9.48±1.95
**Succinic acid**	1010	1.07±0.26	0.22±0.13	1.79±0.43	3.07±1.81
**Benzoic a cid**	1103	1.84±0.17	0.14±0.05	3.08±0.28	1.95±0.70
**Glycine**	1120	0.44±0.15	0.06±0.02	0.74±0.25	0.84±0.28
**Alanine**	1123	1.98±0.21	0.33±0.11	3.31±0.35	4.60±1.53
**Valine**	1268	0.20±0.06	0.05±0.02	0.33±0.10	0.70±0.28
**Leucine**	1354	0.29±0.1	0.06±0.02	0.48±0.17	0.84±0.28
**γ -aminobutyric acid**	1364	3.27±0.04	0.53±0.11	5.47±0.07	7.39±1.53
**Iso-leucine**	1371	0.32±0.06	0.13±0.05	0.54±0.10	1.81±0.70
**Threonine**	1377	0.38±0.11	0.1±0.01	0.64±0.18^b^	1.39±0.14^a^
**Malic acid^b^**	1389	16.35±3.03	1.68±0.46	27.34±5.07	23.43±6.42
**Asparagine**	1411	3.23±0.46	0.22±0.07	5.40±0.77	3.07±0.98
**Proline**	1415	18.28±1.36	1.89±0.05	30.56±2.27	26.36±0.70
**Aspartic acid**	1479	1.30±0.01	0.10±0.02	2.17±0.02^a^	1.39±0.28^b^
**Citric acid**	1488	1.64±0.08	0.43±0.10	2.74±0.13 ^a^	6.00±1.39^b^
**Serine^b^**	1549	0.08±0.02	0.01±0.01	0.13±0.03	0.14±0.14
**Glutamine**	1561	2.82±1.97	0.14±0.02	4.71±3.29	1.95±0.28
**Methionine**	1641	0.06±0.02	0.01±0.01	0.10±0.03	0.14±0.14
**Cystein**	1740	0.01±0.01	0.01±0.01	0.02±0.02	0.14±0.14
**Phenylalanine**	1775	0.12±0.01	0.03±0.02	0.20±0.02	0.42±0.28
**Lysine**	2035	0.39±0.13	0.07±0.02	0.65±0.22	0.98±0.28
**Tyrosine**	2169	0.24±0.03	0.04±0.01	0.40±0.05	0.56±0.14
**Tryptophane**	2332	0.01±0	0.01±0	0.02±0	0.14±0.00

All compounds were identified by matching their retention times and mass spectra with authentic standards. The percentages were calculated by dividing the concentration of each metabolite by the concentration of the total detected compounds then multiplying by 100. Values are given as mean ± SD (n = 5). Different letters indicate statistically significant differences between extraction methods (*P*<0.05).

**Table 2:** Amino acids and organic acid detected in pineapple sweet orange phloem sap by GC-MS after MTBSTFA derivatization (n = 5).

Compound	LRI	Centrifuge-exudate (mM)	EDTA-enhanced exudates	Centrifuge-exudate (%)	EDTA-enhanced exudates (%)
			(m mole/Kg tissue)		
**Alanine**	1534	0.18±0.08	0.02±0.00	0.14±0.06^a^	0.03±0.00^b^
**Glycine**	1561	0.25±0.05	0.14±0.03	0.19±0.04	0.23±0.05
**Valine**	1647	0.85±0.14	0.24±0.09	0.64±0.11	0.40±0.15
**Leucine**	1683	0.53±0.30	0.46±0.15	0.40±0.23	0.77±0.25
**Isoleucine**	1712	0.56±0.13	0.24±0.09	0.42±0.10	0.40±0.15
**Maleic**	1733	0.22±0.06	0.06±0.03	0.17±0.05	0.10±0.05
**Succinic**	1747	1.12±0.14	0.19±0.01	0.85±0.11^a^	0.32±0.02^b^
**Proline**	1754	49.91±5.99	12.90±2.90	37.71±4.53^a^	21.62±4.86^b^
**Fumaric acid**	1782	33.77±5.32	10.51±2.33	25.51±4.02 ^a^	17.61±3.90 ^b^
**Asparagine**	1928	13.28±2.95	9.79±3.51	10.03±2.23	16.41±5.88
**Glutamine**	1940	4.44±1.07	4.07±0.59	3.35±0.81^a^	6.82±0.99 ^b^
**Methionine**	1952	0.47±0.14	0.28±0.06	0.36±0.11	0.47±0.10
**Serine**	1963	9.29±2.19	3.58±0.84	7.02±1.65	6.00±1.41
**Threonine**	1991	1.33±0.13	0.88±0.08	1.00±0.10^a^	1.47±0.13^b^
**Phenylalanine**	2072	0.88±0.14	0.73±0.08	0.66±0.11 ^a^	1.22±0.13^b^
**Malic acid**	2081	3.60±2.10	4.38±1.91	2.72±1.59	7.34±3.20
**Aspartic acid**	2118	4.03±1.29	1.03±0.26	3.04±0.97	1.73±0.44
**Cysteine**	2165	0.25±0.06	0.05±0.01	0.19±0.05	0.08±0.02
**Glutamic acid**	2218	0.73±0.65	0.03±0.00	0.55±0.49	0.05±0.00
**Arginine**	2372	0.26±0.14	0.37±0.20	0.20±0.11	0.62±0.34
**Citric**	2450	5.87±1.08	9.50±3.02	4.43±0.82	15.92±5.06
**Tyrosine**	2474	0.54±0.15	0.22±0.05	0.41±0.11	0.37±0.08

All compounds were identified by matching their retention times and mass spectra with authentic standards. The percentages were calculated by dividing the concentration of each metabolite by the concentration of the total detected compounds then multiplying by 100. Values are given as mean ± SD (n = 5). Different letters indicate statistically significant differences between extraction methods (*P*<0.05).

**Table 3:** Sugars and other metabolites detected in phloem sap by GC-MS after TMS derivatization (n = 5).

Compound	LRI	Centrifuge-exudate	EDTA-enhanced exudate	Centrifuge-exudate	EDTA-enhanced exudate
		(mM)	(m mole/Kg tissue)	(%)	(%)
**Proline**	1318	67.80±10.18	13.83±0.14	19.83±2.98^a^	9.25±0.09^b^
**Glycine**	1325	3.58±0.34	0.33±0.23	1.05±0.10^a^	0.22±0.15^b^
**Succinic acid**	1336	0.75±0.15	0.81±0.23	0.22±0.04^b^	0.54±0.15^a^
**Fumaric acid**	1348	5.31±0.52	0.22±0.01	1.55±0.15^a^	0.15±0.01^b^
**Serine**	1369	11.26±3.68	2.69±0.38	3.29±1.08	1.80±0.25
**Threonine**	1400	2.29±1.07	1.12±0.49	0.67±0.31	0.75±0.33
**Malic acid**	1499	55.06±5.31	21.55±4.11	16.11±1.55	14.41±2.75
**γ-aminobutyric acid**	1558	37.21±2.65	12.78±1.65	10.88±0.78	8.55±1.10
**Threonic acid**	1578	5.97±0.45	3.24±0.36	1.75±0.13	2.17±0.24
**Citric acid**	1859	28.18±3.93	11.49±1.19	8.24±1.15	7.68±0.80
**Quinic acid**	1906	16.37±1.08	10.47±2.23	4.79±0.32	7.00±1.49
**Fructose**	1915	10.33±0.60	9.00±1.11	3.02±0.18^b^	6.02±0.74^a^
**Mannose**	1929	0.71±0.18	0.2±0.24	0.21±0.05	0.13±0.16
**Galactose**	1937	0.53±0.04	0.2±0.24	0.16±0.01	0.13±0.16
**Glucose**	1941	20.47±1.37	23.1±1.52	5.99±0.40^b^	15.45±1.02^a^
Unknown inositol isomer 1*	1997	2.94±0.40	0.97±0.10	0.86±0.12	0.65±0.07
**Palmitic acid**	2043	2.8±1.26	0.118±0.069	0.82±0.37	0.08±0.05
Unknown inositol isomer 2*	2055	0.33±0.04	0.30±0.11	0.10±0.01	0.20±0.07
**Inositol**	2105	2.42±0.09	1.53±0.24	0.71±0.03	1.02±0.16
**Oleic**	2171	0.62±0.032	0.01±0.00	0.18±0.01^a^	0.01±0.00^b^
**Stearic**	2186	1.05±0.52	0.08±0.00	0.31±0.15	0.05±0.00
**Sucrose**	2574	65.89±4.05	35.51±0.42	19.27±1.18 ^b^	23.74±0.28 ^a^

All compounds were identified by matching their retention times and mass spectra with authentic standards. The percentages were calculated by dividing the concentration of each metabolite by the concentration of the total detected compounds then multiplying by 100.^ *^Quantified relative to inositol. Values are given as mean ± SD (n = 5). Different letters indicate statistically significant differences between extraction methods (*P*<0.05).

#### 1-Amino acids

The MCF method was used to measure the concentration of amino acids and organic acids in the phloem sap because MCF does not react with the hydroxyl group of sugars [24]. The MTBSTFA method was used to confirm the results obtained by the MCF method and to determine the concentration of arginine that cannot be detected after MCF derivatization [25]. Eighteen amino acids were identified in the phloem sap using GC-MS after MCF derivatization (Table 1). The concentrations of these amino acids are shown in Table 1. Proline, alanine, asparagine, aspartic acid, and glutamine were abundant in the phloem sap. Gamma–aminobutyric acid (GABA) was the only non-protein amino acid detected, and its concentration in pure phloem sap was about 3 mM (Table 1). The total amino acid concentration in pure phloem sap measured by the MCF method was 33.4 mM (Table 1).

All of the amino acids detected after MCF derivatization (Table 1), except GABA, lysine, and tryptophan, were also detected after MTBSTFA derivatization (Table 2). In addition, two more amino acids were detected (glutamic acid and arginine) after MTBSTFA derivatization (Table 2). The total amino acid concentration in the phloem sap measured by this reagent was about 84 mM (Table 2). Proline was the predominant amino acid in the phloem sap derivatized with MCF and MTBSTFA (Table 1 and Table 2), and its concentration was about 18 mM and 50 mM, respectively.

Proline, glycine, serine, and threonine were also detected in the phloem sap after TMS derivatization (Table 3). In agreement with MCF and MTBSTFA results, proline was the predominant amino acid and its concentration was about 68 mM. GABA was also detected after TMS derivatization (Table 3) and its concentration was about 37 mM. The total amino acid concentration in the phloem sap measured after TMS derivatization was about 122 mM. Histidine was detected in the citrus phloem sap after MCF derivatization (RT = 26.1, LRI = 2087), however its concentration was below the level of quantification. The concentration of total amino acid in the pure phloem sap measured using the ninhydrin method was close to that obtained by GC-MS. Overall; 20 amino acids were detected in the phloem sap by GC-MS. Their proportions to each other are present in Figure 1A-D. Proline was the most abundant amino acid (37–60%) (Figure 1A-D). Asparagine was the second most predominant amino acid with 6–27% of the total amino acids. All of the amino acids that have been reported in citrus juice [23], [26] were also detected in the phloem sap of pineapple sweet orange. Histidine is present in the phloem sap but below the level of quantification. In fact, histidine is present in low amounts in citrus juice [23] and its LOD is usually higher than most of the rest of amino acids [24], [25]. In agreement with our results, proline was the most predominant amino acid in Valencia sweet orange juice [23]. Arginine has also been reported in citrus juice [23], [26]. Arginine was not detected in the citrus phloem sap after MCF derivatization due to the low reactivity of the guanidine group or to the thermal instability of the MCF derivative of this group [25]. On the other hand, small amounts of arginine were detected in the phloem sap after MTBSTFA derivatization. GABA has also been reported in Valencia sweet orange juice [23] and in the phloem sap of many plants such as tomato [27], maize [28], beans, clover alfalfa, and pea [29]. In plants, GABA is synthesized through the shunt metabolic pathway and it accumulates in response to biotic and abiotic stresses [30]. GABA is also an important neurotransmitter widely present in the nerve systems of mammals, insects, round worms, and platyhelminthes [30], [31]. The presence of tricarboxylic acid (TCA) cycle genes in *C*Las genome indicated that *C*Las can use a wide range of amino acids to produce energy [32]. *C*Las can metabolize glutamate, alanine, aspartate, glycine, serine, threonine, methionine, cysteine, arginine, proline, histidine, tyrosine, phenylalanine, and tryptophan [32]. All of the previous amino acids were detected in the phloem sap. Genome sequencing of *C*Las also revealed that *C*Las is not able to synthesize tryptophan, tyrosine, leucine, isoleucine, and valine from metabolic intermediates [32]. Our results showed that those amino acids were present in the phloem sap of sweet orange so they may be used directly by *C*Las. Proline could play an important role in host-pathogen interactions. In fact, many studies showed that some types of bacteria such as *Photorhabdus* spp. and *Xenorhabdus* spp. can sense the presence of proline in insect hemolymph, leading to the activation of various virulence factors and metabolic shift [33], [34]. Proline could also play an important role in citrus–*C*Las interactions. Previous studies showed that the levels of proline, serine, and aspartic acid were higher in HLB-sensitive cultivars [35]. Although 20 amino acids were detected in the phloem sap, only 7 of them (iso-leucine, leucine, valine, lysine, phenylalanine, methionine, and threonine) are considered essential for phloem sap-sucking insects [2]. The percentage of these essential amino acids to total amino acids ranged from 4.1% to 10.0% (Figure 1A–D). The ratio of essential amino acids to non–essential amino acids found in the citrus phloem sap (1∶4−1∶20) is in agreement with those reported in the literature [2]. Because of the low amount of essential amino acids in plant phloem sap, insects depend on the symbiotic bacteria as a main source for those amino acids [2]. Besides being used in protein synthesis, amino acids ingested from the phloem sap are an important source of energy. Amino acids can be converted to trehalose, which is the major source of energy during insect flight [2]. Oxidation of proline has been found to be the major source of fuel for flight muscles in some insects [36].

#### 2- Organic acids

Many organic acids were detected in the phloem sap. Maleic, fumaric, succinic, malic, benzoic, and citric acids were detected in the phloem sap derivatized after MCF derivatization (Table 1). The same acids except benzoic acid were detected after MTBSTFA (Table 2).

Malic acid was the most predominant organic acid in MCF and TMS and its concentration in the pure sap was about 16.4 mM and 55 mM, respectively. Fumaric acid was the major organic acid in the MTBSTFA method and its concentration was about 33.7 mM (Table 3). The total concentrations of organic acid in the pure sap were 30, 89, and 44.6 mM after MCF, TMS, and MTBSTFA derivatization, respectively. Figure 3 illustrates the proportions of the organic acids to each other in the three derivatization methods and the two phloem sap collection methods (Figure 2A–F). The high levels of these organic acids may contribute to the acidity of the phloem sap.

Citrate, malate, succinate, tartaric, benzoic, oxalic, ascorbic, and lactic acids were reported in orange juice [23], [37], [38], [39]. Whereas malic acid was the most predominant organic acid in the phloem sap derivatized with TMS and MCF (Figure 2A–D), fumaric acid was the highest after MTBSTFA derivatization (Figure 2E–F). Heat applied during MTBSTFA derivatization could be the reason behind the increase in fumaric acid content in the phloem sap. Previous studies showed that heat induces the formation of fumaric acid in apple juice [40]. Malic, maleic, fumaric, succinic, citric, and fumaric acids are intermediates of the TCA cycle [41] and could be directly integrated in the TCA cycle as a source of energy of *C*Las. Because the isocitrate lyase and malate synthase are absent in the *C*Las genome, *C*Las uses exogenous fumarate, malate, succinate, and aspartate as carbon substrates for the TCA cycle and pyruvate generation [42]. Malic, maleic, citric, succinic, and fumaric acids were abundant in citrus phloem sap. Because these compounds are important intermediates in the TCA cycle in insect [43], they could also be incorporated in the TCA cycle to produce energy. In addition, malate, fumarate, succinate, and citrate acids play an important role in proline metabolism and its synthesis from alanine in insects [44].

#### 3- Sugars

The TMS silylation method was selected to analyze the sugar content of the phloem sap because it results in stable and reproducible derivatives for a wide range of sugars and organic acids [45]. Sucrose, fructose, glucose, trace amounts of mannose, and galactose, and three sugar alcohols were detected in the phloem sap after TMS derivatization (Table 3). Sucrose was the most abundant sugar and its concentration was about 66 mM. Glucose and fructose were the most predominant monosacharides, and their concentrations were 20 and 10 mM, respectively. The total sugar concentration in the phloem sap was about 103 mM (Table 3). As proportions, sucrose, glucose, and fructose were the major sugars in the sap and they comprised about 64%, 20%, and 10% of the total sugars, respectively (Figure 3A–B). Sugar alcohols made up about 5% of the total sugars (Figure 3A–B). The sugars detected in the phloem sap are also abundant in sweet orange juice but their concentrations in juice were higher [21], [23]. CLas depends on the available nutrients in the phloem sap [23] and it could use these sugars to produce energy. In fact, sequencing of CLas showed that glycolysis was the major pathway for the catabolism of monosaccharides and it also showed that CLas could metabolize glucose, fructose, and xylose [32]. In addition, phloem sap-sucking insects break down sucrose to glucose and fructose and use them as a source of energy [2]. Glucose and fructose could also be converted to trehalose, which is used as a source of energy in insect muscles during flight [44]. Three sugar alcohols were detected in the phloem sap; inositol and two unknown inositol isomers (Figure 2A–B). In general, inositol plays an important role in various biological processes in plants [46]. Scylloinositol and its precursor myo-inositol have been found in the hemolymph of insects [47]. Inositol has been found to be an important requirement for immature stages of some hemimetabolan insects such as locusts [48].

#### 4- Fatty acids

Low quantities of palmitic acid, oleic acid, and stearic acid were detected in the phloem sap after TMS derivatization. The concentrations of these fatty acids in pure phloem sap ranged from 0.6 to 2.8 mM (Table 3). The total fatty acid concentration in the phloem sap was less than 5 mM. Although most of the earlier studies did not focus on the lipid composition of the phloem sap, recent studies showed that fatty acids are present [49], [50]. Palmitic, oleic, and stearic acids were the most predominant fatty acids in canola and Arabidopsis phloem sap [49], [50]. Similar to phloem sap, trace amounts of lipids (840 to 1010 ppm) were detected in citrus juices, and palmitic and oleic are among the most predominant fatty acids in orange juice [51]. Although CLas cannot grow on fatty acids [42], it is not clear if these fatty acids are essential for CLas pathogenicity. Similarly, feeding studies showed that phloem sap-sucking insects such as aphids can be reared on a fat-free diet for several generations, which means that aphids are able to make their essential fatty acids. In addition, aphids treated with antibiotics were able to synthesize linoleic acid, which indicated that linoleic acid synthesis is independent of aphid's symbionts [52].

## Conclusion

The phloem sap of sweet orange is rich in sugars, amino acids, and organic acids. In general, all the metabolites detected and their percentages in phloem sap collected by EDTA exudation were similar to the sap collected by centrifugation. However, the centrifugation method allowed us to estimate the concentrations of different compounds. The percentages of chemical groups using the different derivatization methods are presented in Figure 4A–C. Most of the metabolites found in phloem sap are also detected in citrus juice; thus, it is not surprising that the addition of citrus orange juice to the culture medium prolonged the viability of *C*Las [53]. The phloem sap is considered an excellent diet for phloem-sap feeders because it is predigested, rich in sugars and amino acids, and it is free of toxins and feeding deterrents [2]. This study revealed aspects of the nutrient composition of the citrus phloem sap that could contribute to the cultivation of *C*Las and formulation of an artificial diet system for phloem sap-sucking insects that attack citrus.

## Material and Methods

### Materials

Sucrose, glucose, fructose, mannose, galactose, malic acid, inositol, citric acid, quinic acid, benzoic acid, fumaric acid, glycine, alanine, valine, leucine, isoleucine, theronine, proline, glutamine, methionine, cystein, histidine, tyrosine, arginine, lysine, asparagine, aspartic acid, phenylalanine, glutamic acid, serine, threonine, tyrosine, γ-aminobutyric acid, methoxyamine hydrochloride solution (MOX) in pyridine (2%), N-methyl-(N-trimethylsilyl) trifluoracetamide (MSTFA), methylchloroformate (MCF), sodium hydroxide, pyridine, methanol, N,N-dimethylformamide, chloroform, sodium bicarbonate, and sodium ethylenediaminetetraacetic acid (EDTA) were purchased from Fisher Scientific (Pittsburg, PA, USA). Hydrindantin, ninhydrin, lithium hydroxide, and N-methyl-N- [tert-butyl dimethylsilyl]-trifluroacetamide (MTBSTFA), and amino acid standard mix were purchased from Sigma-Aldrich (St. Louis, MO, USA).

### Plants

We used Pineapple sweet orange (*Citrus sinensis* (L.) Osbeck) plants (12 months old, 0.75–1 m tall). Plants were kept in a USDA-APHIS approved secure greenhouse with the temperature controlled at 28–32°C. Plants were regularly watered twice a week and fertilized once a week using 20-10-20 fertilizer (Allentown, PA, USA).

### Phloem sap collection

#### 1- Centrifugation technique

Stems of 10–20 cm (0.5 cm diameter) were collected from one-year-old greenhouse plants. The bark was stripped into two pieces and was manually removed from the twig. The inner part of the bark was rinsed with deionized water and dried with Kim wipes to exclude any contamination from the xylem sap. Then the bark strips were cut into about 1-cm pieces using a sterile razor blade. To collect the phloem sap, three to five pieces of the bark tissue were vertically placed in a 0.5-ml Eppendorf tube. A small hole was made at the bottom of the tube using a razor blade and the tube was immersed into a 2-ml Eppendorf tube. The sample was centrifuged at 12,000 rpm for 15 min at room temperature and the collected phloem sap was stored at −80°C until analysis. Collected pure phloem sap from five plants was pooled together and considered as one replicate. Five replicates were used for the GC-MS analysis.

#### 2- EDTA-enhanced exudation technique

The phloem sap of pineapple sweet orange was also collected using ethylenediaminetetraacetic acid (EDTA) as described by Rennenberg et al. [4]. Briefly, 100 mg of pineapple bark were exudated with 1 ml of 5 mM EDTA (pH 7.0.) for 5 h at room temperature. Phloem sap exudates from five plants was pooled together and considered as one replicate. Five replicates were used for GC-MS analysis.

### Measurement of pH, °Brix value, and total amino acids

The pH of the sweet orange pineapple phloem sap, obtained by centrifugation or EDTA–enhanced exudation technique, was measured using an Orion RSS® micro pH meter electrode (Pittsburg, PA, USA).

The moisture content was determined by drying the phloem tissue samples to a constant weight at 100°C. The °Brix values were measured using Mark II digital Refractometer (Reichert, Inc., Depew, NY, USA). The °Brix values of pure phloem sap obtained by centrifugation were measured directly. To estimate the °Brix value in the phloem sap collected by the EDTA method, 1 ml (obtained from 100 mg fresh weight) was concentrated under nitrogen stream to 75 µl (water content in 100 mg fresh weight tissue). The total amino acids in the phloem sap were measured with ninhydrin [54].

### Phloem sap derivatization

#### 1- Methylchloroformate (MCF) derivatization of amino acids

The amino acids in the phloem sap were derivatized with MCF and analyzed with GC-MS [55]. Briefly, a 20-µl aliquot of the pure phloem was transferred to a 1-ml silanized GC-MS insert and mixed with 200 µl of NaOH (1 M). Then the alkaline sample was mixed with 167 µl of methanol and 34 µl of pyridine, followed by the addition of 20 µl of MCF. The sample was vigorously mixed for 30 s. An additional 20 µl of MCF was added and the sample was mixed for another 30 s. A 200-µl aliquot of chloroform was added with vigorous mixing for 10 s, followed by 200 µl of sodium bicarbonate (50 mM) with vigorous mixing for 10 s. The upper layer was discarded and approximately 100 µl of the organic layer was transferred to a new insert. A few milligrams of sodium sulfate were added to dry the organic layer, and 0.3 µl was injected into the GC-MS. A 1-ml aliquot of the EDTA exudates (equivalent to 0.1 g phloem sap fresh weight) was concentrated to 20 µl under nitrogen stream and derivatized as mentioned above. A 40-, 20-, 10-, and 5-µl aliquot of 1000 ppm of amino acid standard mixture was derivatized as described above and used to calculate the amino acid concentration in the phloem sap.

#### 2- N-methyl-N- [tert-butyl dimethylsilyl]-trifluroacetamide (MTBSTFA) derivatization

A 25-µl aliquot of the pure phloem sap or 1 ml of EDTA exudates was dried under nitrogen stream and derivatized with MTBSTFA [9]. Briefly, the dried samples were mixed with 100 µl of N,N-dimethylformamide and 50 µl of MTBSTFA and derivatized by heating at 80°C for 45 min. A 0.5-µl aliquot of the derivatized samples was injected into the GC-MS running in full scan mode. A 40-, 20-, 10-, and 5-µl aliquot of 1000 ppm of amino acid standard mixture was derivatized in the same way and used to calculate the amino acid concentration in the phloem sap.

#### 3- TMS-derivatization of sugars

A 5-µl aliquot of the pure phloem sap or 1 ml of EDTA exudates (equivalent to 0.1 g fresh weight phloem sap) was transferred to 2-ml micro-reaction vessel and dried under nitrogen stream. The dried sample was mixed with 30 µl of methoxyamine hydrochloride solution (MOX) in pyridine (2%) and allowed to react for 17 h at room temperature [56]. At the end of the methoximation, the sample was mixed with 80 µl of N-methyl-(N-trimethylsilyl) trifluoracetamide (MSTFA) and left for 2 h at room temperature. Finally, 0.3 µl of derivatized sample was injected into the GC-MS running in full scan mode. Standard mixes (sucrose, glucose, mannose, fructose, galactose, inositol, malic acid, quinic acid, and citric acid) were processed as described above and used to quantify the concentration of phloem sap components.

### GC-MS analyses

Derivatized samples and standards were analyzed using a Clarus 500 GC-MS system (Perkin Elmer, Waltham, MA, USA) fitted with an HP-5MS column (cross-linked 5% Ph Me siloxane, 50 m×0.22 mm×0.025 µm film thickness). The flow rate for the hydrogen carrier gas was 1 ml/min. The GC temperature program was as follows: initial temperature was held at 70°C for 5 min, and then increased to 180°C at a rate of 10°C/min, held for 2 min, increased further to 280°C at 10°C/min, held for 1 min, increased to 300°C, and finally held for 5 min. The injector and the detector temperatures were set at 220°C and 260°C, respectively.

### MS peak identification

GC-MS chromatograms were analyzed using TurboMass software version 5.4.2 (Perkin Elmer, Waltham, MA, USA). Peaks were first identified by comparing their mass spectra with library entries (NIST mass spectra library (National Institute of Standards and Technology, Gaithersburg, MA, USA); Wiley 9th edition (John Wiley and Sons, Inc., Hoboken, NJ, USA)). Identification of some compounds was further confirmed by comparing their retention time and mass spectra with authentic standards. In addition, the linear retention indices (LRI) of the detected compounds were calculated using a calibration curve generated by injecting a mixture of alkane (C8–C18).

### Statistical analysis

The percentage of each component in the phloem sap obtained by EDTA-enhanced method and centrifugation method was calculated by dividing its concentration by total concentration of all detected components. Two tail t-test were used to compare the percentage of each component in both phloem sap collecting methods.
